# Preliminary investigation of the effects of topical mixture of *Lawsonia inermis* L. and *Ricinus communis* L. leaves extract in treatment of osteoarthritis using MIA model in rats

**DOI:** 10.1186/s40199-016-0152-y

**Published:** 2016-05-03

**Authors:** Atousa Ziaei, Shamim Sahranavard, Mohammad Javad Gharagozlou, Mehrdad Faizi

**Affiliations:** Traditional Medicine and Material Medical Research Center; Department of Traditional Pharmacy, School of Traditional Medicine, Shahid Beheshti University of Medical Sciences, Tehran, Iran; Department of Pathology, Faculty of Veterinary Medicine, University of Tehran, Tehran, Iran; Department of Pharmacology and Toxicology, School of Pharmacy, Shahid Beheshti University of Medical Sciences, Tehran, Iran

**Keywords:** *Lawsonia inermis* L, Leaves, MIA, Osteoarthritis, Rats, *Ricinus communis* L

## Abstract

**Background:**

Many plants have been introduced in Iranian traditional medicine for treatment of different joint problems including knee pain. Topical application of the mixture of *Lawsonia inermis* L. leaves (Henna) with aqueous extract of *Ricinus communis* L. leaves have been mentioned to have significant effects on reducing knee pain. The present study was designed to evaluate the analgesic and anti-inflammatory effects of the mixture of these two herbs in male rats.

**Methods:**

We induced knee osteoarthritis as a model of chronic pain by intra-articular injection of mono sodium iodoacetate (MIA). Mechanical allodynia, hotplate latency test, spontaneous movements and gait analysis were used for the evaluation of analgesic activity. Anti-inflammatory activity was evaluated by measuring the diameter and the volume of the injected paw compared to contralateral paw. These tests were monitored at days 1, 3, 7, 14 and 21 of MIA administration. Histopathological evaluations were also used to assess the efficacy of the treatment on inflammation and lesions in knee tissue. In all tests, diclofenac topical gel was used as a positive control. The herbal extracts, their mixture, and vehicle or diclofenac gel were administered daily for 14 days by topical route.

**Results:**

The mixture of these two extracts significantly reduced the knee joint width and volume of the injected paws and also improved foot prints in gait analysis after 3 days of MIA injection. Analysis of mechanical allodynia (after 21 days), hotplate latency test (after 10 days), spontaneous movements (after 7 days) and in positive control group (after 3 days in all tests and in mechanical allodynia after 14 days) compared to the vehicle group, showed significant effects. Topical usage of the selected formulation made significant histopathological changes on the knee of the rats. Compared to the vehicle group, the tests and diclofenac groups showed less reactions characterized by negligible edema and a few scattered inflammatory lymphoid cells.

**Conclusion:**

The present findings showed that the present formulation not only was able to mitigate pain and inflammation in the paws but also made significant histopathological changes on the knee of the rats. Further studies are necessary to confirm the effect of the formulation.

## Background

Pain is a major symptom in many medical conditions and it is the most common reason for medical consultation. Approximately half of all licensed drugs that had been registered worldwide in a 25 years period prior to 2007 were natural products or synthetic derivatives of natural products [1]. For thousands of years, natural products derived from plants, animals and microorganisms have been used as treatments for human diseases. Knowledge of the medical use of natural products has been transmitted from generation to generation over the years [2]. It seems that drugs, especially those which have plant origin and have been used in the Iranian traditional medicine (ITM) could be an appropriate initiative in research projects aiming at development of new analgesic drugs.

A formula containing the mixture of *Lawsonia inermis* L*.* and *Ricinus communis* L. was chosen for evaluation of knee pain reduction from the Iranian traditional medicine books such as: *Makhzan-ol Advieh* [3], *Gharabadin-e-kabir* [4] and *Tohfat al-mu’minin* [5]. In these books, the mixture of mentioned plants has been recommended for knee pain treatment.

Osteoarthritis (OA) is a degenerative joint disease characterized by joint pain and progressive loss of articular cartilage [6]. Mono sodium iodoacetate (MIA) is a chemical substance that induced OA as a model of knee pain. This model is used for the study of pain and analgesic drug effects because it is reproducible and mimics the pathological changes and the pain of osteoarthritis in humans [7]. Injection of MIA, an inhibitor of glycolysis, into the femorotibial joint of rodents, promotes loss of articular cartilage similar to that observed in human OA [6]. The joint problem induced in this way, is called induced MIA hereafter.

*Lawsonia inermis* L*.* (Lythraceae) is used in the treatment of diseases such as leprosy and headache and has cosmetic purposes like accelerating the growth and dying hair and nails [8] and has also been reported to have anti-inflammatory, antinociceptive and antipyretic effects [9]. The natural constituents of *L.inermis* are Lawsone (2-hydroxy-1,4-naphthoquinone), mucilage, essential oils, tannic acid, gallic acid, fats, glucose, mannitol, and resin [10].

*Ricinus communis* L. (Euphorbiaceae) is used for the treatment of swelling, gout and skin diseases [11]. Polyphenols and flavonoids are the major compounds found in this plant and have anti-inflammatory and antioxidant activities [11, 12].

The present study was designed to evaluate the analgesic and anti-inflammatory effects of the mixture of topical extracts of *L.inermis* and *R.communis* according to the Iranian traditional manuscripts. The efficacy of the used formulation in reducing knee pain was evaluated by inducing osteoarthritis. All of the pharmacological experiments were also performed to determine the effects of *L.inermis* or *R.communis* extract separately.

## Methods

### Preparation of the extracts

Dry leaves of *Lawsonia inermis* L*.* were gathered from Yazd, Iran and identified and authenticated by a plant taxonomist at the herbarium of School of Traditional Medicine, Shahid Beheshti University of Medical Sciences, Tehran, Iran. The voucher specimen was deposited with number of HMS 331 in the herbarium. The dried leaves were powdered coarsely with a mechanical grinder (Desktop mill, 8300, Iran). The powder was passed through sieve No.40 and stored in an airtight container for further use. The powdered leaves were macerated in ethanol and water (80:20) and allowed to shake for 24 h and then were filtered through a filter paper (Whatman filter paper No.1). The maceration process was repeated 3 times. The filtered extract was concentrated in a rotary evaporator (Heidolph, HB digital, Germany) at 40 °C and then used freeze drier (Benchtop, SLC Virtis, USA) to remove water (the herbal extract ratio was 19 %). The dry extract was stored in cool place until used.

Fresh leaves of *Ricinus communis* were gathered from Tehran, Iran and identified and authenticated and deposited similar to *L.inermis* with voucher number of 3577. Subsequently, leaves were dried in shade and powdered by a mechanical grinder. The powdered leaves were macerated in water and allowed to shake for 24 h, same as described for *L.inermis* (the herbal extract ratio was 22 %). The herbal extracts were added at the same concentrations as mentioned in ITM references [3–5] and we did not perform comparative evaluation of different doses of the extracts.

### Drug administration

The dry extracts were mixed with the same percentage, suspended in water (0.2 g/0.3 ml). This is the maximum amount of the mixture of extracts that solved in the lowest amount of the vehicle and cover all of the animal ’s knee and administered topically on the left hind paw of the animal in all groups, from day 1 to 14 (once a day dosing) respectively. The control group (Vehicle) received the same volume of vehicle (water) by the same route. 0.4 g of diclofenac 1 % gel (Razak Pharmaceutical Company) was used topically in the positive control group [13].

### Animals and experimental groups

Wistar male rats (160–180 g body weight) were purchased from the Pasteur Institute of Iran. They were housed in standard polypropylene rat cages and kept in a room with controlled condition (temperature 25 ± 2 °C and relative humidity 40–50 %) in a 12 h light-dark cycle. The rats were given a standard laboratory diet and have free access to food and water. Each rat was only used once. All procedures for the treatment of animals were approved by the Research Committee of Shahid Beheshti University of Medical Sciences and institutional animal care and use committee with approval code SBMU.REC.1392.343.

For induction of OA, rats were anesthetized with ketamine-xylazine (100 and 10 mg/kg) intra-peritoneally [14, 15]. Osteoarthritis was induced by an injection of mono sodium iodoacetate (MIA, Sigma-Aldrich, USA) at a dose of 3 mg/50 μL normal saline to intra articular space of the left hind limb [16–18].

In this study, 36 adult male wistar rats were used. They were randomly divided in to 6 groups including sham group (S; *n* = 6) that received saline instead of MIA, negative control group (N; *n* = 6) with OA induction and treating by vehicle, combination group (T; *n* = 6) with OA induction and treating by topical mixture of the plant extracts, *L.inermis* group (L; *n* = 6), *R.communis* group (R; *n* = 6) and positive control group (P; *n* = 6) with OA induction and treating by diclofenac topical gel. On day 21, after performing behavioral tests, rats of all groups were sacrificed by overdosing ether [19]. To evaluate the histological changes, rats were sacrificed on day 14 after 3 h of the formulation or diclofenac gel administration and left knee was collected for histological examination.

### Behavioral tests

#### Mechanical allodynia (Von Frey test)

Von Frey filaments (North Coast Medical, Inc. CA, USA) have been used to assess the mechanical sensitivity of the hind paw of the animals with knee joint arthritis. Typically, paw withdrawal threshold (PWT) is measured in response to increasing pressure stimuli applied to the plantar surface by von Frey filaments. Rats were removed from their home cages and placed in a Plexiglass cage with a wire mesh bottom [20]. The rats were allowed to acclimate for 15 min (or until exploratory and grooming behavior declined to a level compatible with behavioral testing). Von Frey monofilaments were applied at a 90° to the mid-plantar of the left hind paw of the rats (ipsilateral side of MIA injection) with a series of monofilaments that ranged from 0.6 to 26 g in stiffness. Filaments were held in place and then removed; they were applied at the same location; 5 times for 1.5 s with inter stimulus intervals of 1 min. Rats were tested using the up-down method. A positive response was defined as a rapid withdrawal of the left hind paw or licking of the paw (three out of five were considered positive). The first day of the testing provided a baseline measure of tactile sensitivity. Rats in each group were then tested with von Frey monofilaments on post-injection days 1, 3, 7, 10, 14 and 21.

#### Spontaneous locomotor activity (Open field test)

This method has been used to evaluate the locomotor activity of rodents [21]. The test was performed in a Plexi glass box of 40 × 40 × 40 cm with transparent walls and black floor (in contrast with the color of the rat). Each rat was initially placed in the center of the box and its activity was recorded by a video camera for 10 min (at the same temperature and light conditions). Locomotor activity was measured in the square arena. The behavior was recorded by a video camera mounted on the ceiling, relayed to a monitor and total distance moved of the rat was analyzed by tracking software (EthoVision, Noldus, The Netherlands). Spontaneous locomotion was assessed on six consecutive days. On each day, each rat was placed in the center of the arena and allowed to explore it for 10 min. In this period, the rat’s movements were recorded with a video camera. The computer software calculated the distance that the rat moved and the total distance during 10 min period was measured. At the end of each test, the box was removed and the entire test chamber was cleaned with a damp cloth and subsequently dried [22].

#### Gait analysis test (Footprint)

Analyzing the walking patterns of the rat by recording its footprints is a well-established and widely employed method for the assessment of motor nerve recovery after nerve injury [23]. We used the software image J to analyze the rat’s footprints. Analysis footprints by image J 1.37 software is extremely useful and reduces intervention results such as operator subjectivity which can limit the statistical significance of the numerical data generated.

Tracking tunnels are basically rectangular, designed to allow the target animal to walk through unhindered. A tracking paper made of an absorbent white paper was used. Rats were stained with the ink-foot stump then each animal attracted into the tunnel after walking across the ink leaves footprints on the absorbent paper. The ink is absorbed into the paper leaving tracks which can be analyzed by the software image J [24].

### Hotplate test

The analgesic response was the latency observed from the time the rat was placed on the heated surface until the first overt behavioral sign of nociception such as (a) the rat licking a hind paw, (b) vocalization or (c) an escape response [25].

Animals were placed individually on a hotplate with the temperature adjusted to 52 °C (UgoBasile, Varese, Italy). Exposure to heat continued until a nociceptive reaction in either of the hind paws occurred. The latency of the withdrawal response of each of the hind paws was determined at 1, 3, 7, 10, 14 and 21 days after injection of MIA. The heat source was maintained at constant intensity, which produced a stable withdrawal latency of approximately 8–10s in vehicle group. The animals were tested in only one series of measurements and the typical responses were hind paw shaking and/or lifting and the rat was immediately removed from the hotplate after the response was observed. The latency to the response was recorded manually with a chronometer and the maximum permanence permitted on the hot surface was 60s. The experiments were performed in a sound-attenuated and air-conditioned (25–30 °C) laboratory.

### Inflammatory tests

#### Measuring paw diameter

The paw diameter was measured at intervals of 1, 3, 7, 10, 14 and 21 days after the injection of MIA using Colis (Helios, Germany) after MIA injection. The difference between inflamed and right knee^,^s joint width at 6 time points was calculated (indicating the degree of inflammation) and was compared to the amount for vehicle group (N) [26].

#### Measuring paw edema using mercury

This method was done by the method previously reported by Fereidoni and his colleagues [27]. A cylinder filled with mercury was placed on a sensitive digital balance. The values on the digital balance were recorded. According to the gravity of mercury, the expected measures were calculated and compared with the observed value. The formula used for this measurement is *V* = *W*/*p*, in which *V* stands for volume, *W* for weight and *p* for gravity [27]. Measurements of the inflamed paw were continued for 21 days after MIA injection and performed five times on each rat and the average of middle three values was calculated.

### Histological evaluation

Histological studies were performed to ensure the responses obtained from the pharmacological experiments. On MIA post day 14, rats were sacrificed and MIA or saline injected knee joints (including distal femur and proximal tibia) were fixed in 10 % buffered formaldehyde solution, decalcified using formic acid-sodium citrate method [28]. This method is superior to other decalcification techniques, since preserves the histological and staining properties of tissues very well. The formalin-fixed specimens were washed properly by distilled water to remove the residues of formaldehyde from the tissues. Then, the specimens were transferred into a jar containing sufficient volume of formic acid–sodium citrate solution, prepared as follows [28].

Solution A: 50 g of sodium citrate was dissolved in 250 ml of distilled water.

Solution B: 125 ml of 90 % formic acid were added to 125 ml of distilled water.

To make a working solution, equal volumes of the solutions A and B were mixed before use. Due to higher volume of the bone tissue of the specimens, the decalcifying solution was changed every single day until decalcification was completed. The decalcified specimens were washed very well with distilled water in order to remove decalcified residues.

A longitudinal section was made at the extensor site in such a manner that divided the specimen into two equal pieces. The tissue samples were processed in a tissue processor, paraffin blocks were made and 5–6 μm thick sections were made with a microtome. Sections were stained with the Harris haematoxylin and eosin method [28]. The histology was evaluated through double-blind observations following the method described previously.

Scoring of the severity of the articular or periarticular tissue lesions including acute or chronic inflammatory lesions in the tissue sections stained with Harris haematoxylin and eosine method was done by using a magnification of X100-X400 as follows:

- **0: Negative;** Normal tissue architecture.
- **1: Mild;** A very mild tissue edema accompanied by a few scattered mononuclear cells including lymphocytes.
- **2: Moderate;** Many mononuclear or polymorphonuclear leukocytes accompanied by hyperemia and edema or beginning of granulation tissue formation.
- **3: Severe;** Marked acute or chronic inflammation characterized by fibrinopurulent exudates or granulation tissue formation accompanied by mononuclear infiltration with or without tendinal adhesions to the adjacent tissues.
- **4: Very severe;** Marked severe acute or chronic inflammation accompanied by tissue necrosis and tendinal adhesions.

The number of pathology sections that were used: three sections in the Sham group, four sections in rats, a day after they were injected with MIA(Inflammation peak), four sections in group (P), four sections in group (T), five sections in group (N) with no treatment.

Histological studies were done on five groups: 1- Sham injected by Saline (group S). 2- One day after injection of MIA that substantial inflammation of the synovial joints was observed in the model. Some days later, the inflammatory response in the synovium subsides, necrotic cartilage collapse, and chondrocytes are lost [29, 30]. 3- Diclofenac gel or group (P) as positive group 4-Mixture of the extracts or group (T) 5- Vehicle or no treatment or group (N).

### Statistical analysis

The obtained data were analyzed by the statistical program Prism 5. Results launched by Average ± SEM. Due to the two interventions (different groups on different days), we used two-way ANOVA to determine the differences between the experimental groups and the mean obtained in the presence of interference interaction between groups. To evaluate the significant differences between the groups, the post hoc Bonferroni test was used. Amounts of *p* < 0.05 were considered as the minimum level of significance. Asterisks indicate a statistically difference from group (N); * *p* <0.05, **p <0.01, ***p <0.001.

## Results

### Mechanical allodynia

**Fig. 1:**
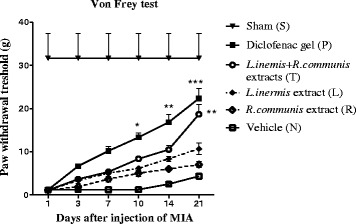
Paw withdrawal threshold (PWT) was measured by von Frey monofilaments. At days 1, 3, 7, 10, 14, 21 after injection of MIA (*n* = 6), von Frey testing on the inflamed paw showed significant effect compared to group (N); after 21 days in test group (T) and after 10 days in positive control or group (P) of MIA injection. Data present mean ± SEM; *p < 0.05, **p <0.01, ***p <0.001 compared to group (N)

Results are expressed as pain threshold measurements with von Frey filament stimulation of the area. A decrease in pain threshold compared to group (N) was demonstrated in all of the groups on the ipsilateral side (Fig. 1).

Treatment was initiated 1 day after the MIA injection and pain was assessed on the days 1, 3, 7, 10, 14 and 21. Paw withdrawal threshold significantly increased (indicating less pain) in the test group and the diclofenac gel group compared with vehicle and sham groups. The pain threshold specifically increased in group (P) after 10 days and in group (T) after 21 days compared to group (N) (*p* < 0.05) (Fig. 1).

### Spontaneous locomotor activity

**Fig. 2:**
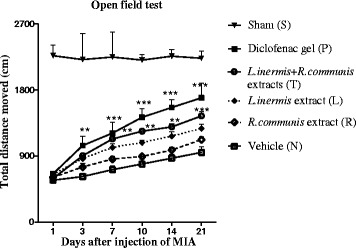
Spontaneous locomotor activity was measured in open field test. Total distance moved of rats in each group was checked. Data were collected over 6 consecutive days (1, 3, 7, 10, 14, 21 after injection of MIA) and averaged per group (*n* = 6). Topical administration of the formulation in group (T) significantly increased the total distance moved after 7 days and in group (P) after 3 days. Data present as mean ± SEM; *p < 0.05, **p < 0.01, ***p < 0.001 compared to group (N)

Total distance traveled in the arena during 10 min showed a significant effect after 7 days compared to group (N). Topical administration of diclofenac gel also significantly increased the total distance moved after 3 days (Fig. 2).

### Footprint

**Fig. 3:**
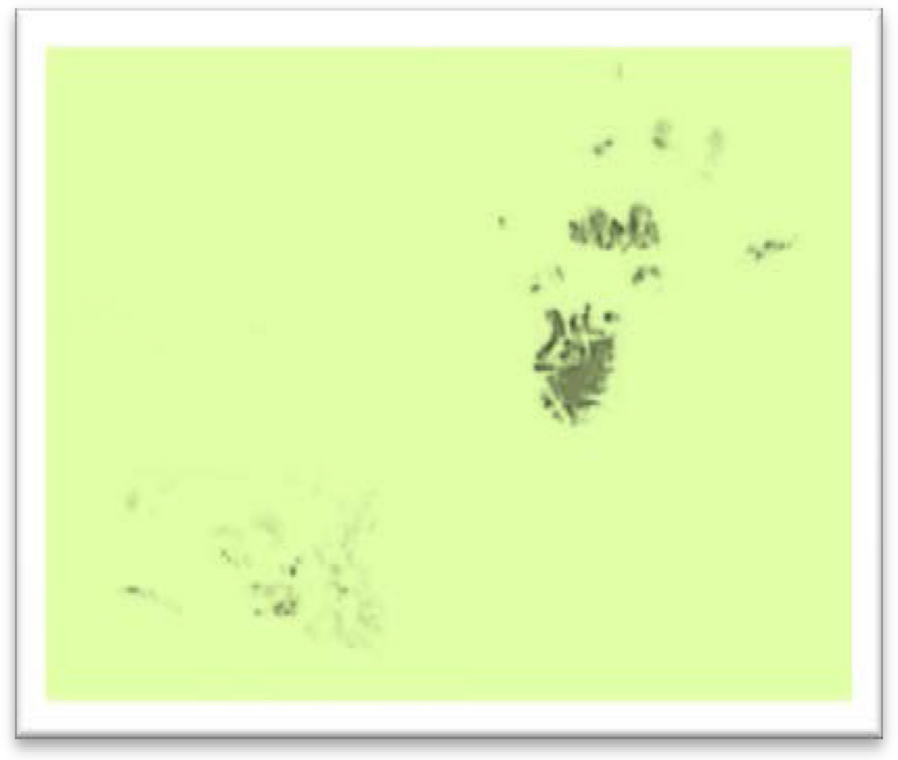
Walking tracks

**Fig. 4:**
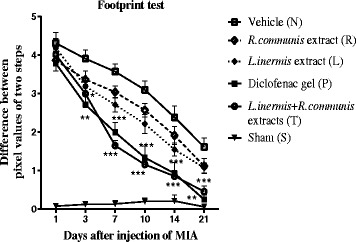
MIA injection affected weight bearing of the paws during locomotion. Comparing the differences between pixel values of right and left hind paw tracks on the paper which obtained by image J software in different days. Significant reduction between pixel values of two steps were seen in group (T) and group (P) after 3 days compared to group (N). Data presented as mean ± SEM; *p < 0.05, **p < 0.01, ***p < 0.001 compared to group (N)

Images of footprint patterns enabled the observation of abnormalities in the foot placing (Fig. 3). The rats in group MIA presented measurable foot placing for 21 days. These animals loaded their weight on the medial part of their affected foot. In group sham, no changes were developed. Significant changes were seen in group (T) and group (P) after 3 days compared to group (N). They were measured both in the affected and in the non affected hind legs (Fig. 4).

### Hotplate

**Fig. 5:**
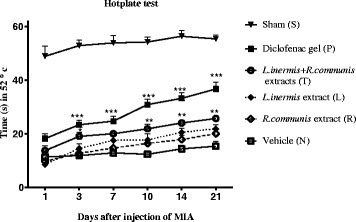
The response latency time of the paws to heat stimulation in hotplate test. The response latency time of the paws was measured at 1, 3, 7, 10, 14, 21 days after injection MIA compared to group (N) in each group. The latency values were increased after 10 days in group (T) and after 3 days in group (P). Data are expressed as mean ± SEM; *p < 0.05, **p < 0.01, ***p < 0.001 compared to group (N)

As illustrated in Fig. 5, the Intra- articular injection into the left hind paw of the rats caused a reduction in the latency of the withdrawal response to heat stimulation compared to sham group (S). The latency values as compared to group (N), was increased after 10 days and in group (P) after 3 days compared to group (N) (Fig. 5).

### Measuring paw diameter

**Fig. 6:**
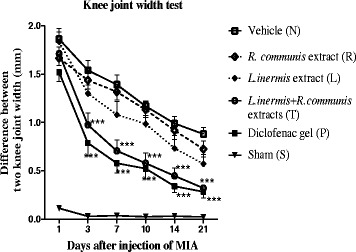
Comparison the differences between the inflamed and right knee^,^s joint width. The knee^,^s joint width was measured by colis in different days 1, 3, 7, 10, 14, 21 for each group. Treatments were continued for 14 days. Compared to group (N), differences between two knee joint widths were significantly reduced in group (T) and group (P) after 3 days. Data are expressed as mean ± SEM; *p < 0.05, **p < 0.01, ***p < 0.001

In Fig. 6, Data of anti-inflammatory activity of the extracts in MIA induced paw edema are shown. The left paw diameter after different day s intervals was used as criteria for evaluation of inflammation. Generally, data indicated that the extracts possessed anti-inflammatory activity compared to the vehicle group and it showed after 3 days significant effect as compared to group (N) can be seen and it was as the same as the effect of group (P) (Fig. 6).

### Measuring paw volume

**Fig. 7:**
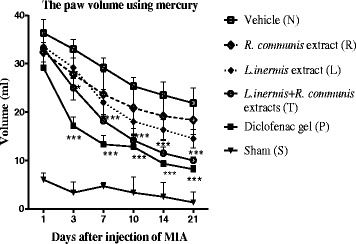
The volume of the inflamed paw was measured with mercury coloumn in different days. The paw volume significantly reduced after 3 days in group (T) and group (P) compared to group (N). Data are showed as mean ± SEM of edema volume induced by MIA; *p < 0.05, **p < 0.01, ***p < 0.001 compared to group (N)

Chronic pain suffering rats were given daily topical administration of extracts, diclofenac and vehicle for 14 days. The volume of the left hind paw was measured with mercury column in different days. The effect was observed after 3 days as the same as diclofenac gel and the paw volume was reduced compared to the vehicle group (Fig. 7).

Topically using *L.inermis* (henna) extract on each paw was effective in reducing the OA pain and inflammation, compare to vehicle but p value was not less than the accepted level of significance (*p* > 0.05) therefore it did not achieve complete pain remission. Topically using *R. communis* was not effective as *L. inermis* and showed little antinociceptive and anti-inflammatory activities in pharmacological tests.

### Histology

**Fig. 8:**
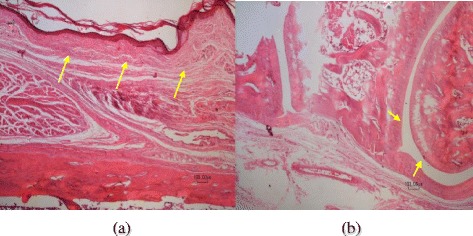
A section of articular tissue from group (S) or sham. The H&E stained paraffin tissue sections of covering skin tissues (*large arrows*) and articular (*small arrows*) from saline injected group can be seen. The skin (**a**) and articular tissue (**b**) have normal tissue architecture. Scale bar = 100 μm (**a**,**b**)

The results of the pathological findings and scoring of the lesions are depicted in Table 1. As seen in Table 1, the Sham group received saline and was euthanized 14 days later. The articular tissue structures including synovium, synovial tissue, adjacent ligaments, tendons and tendinal sheath and subcutaneous tissues and muscles had normal tissue architecture and were intact (score0) (Fig. 8).

**Table 1:** Comparison of the histological effects of different groups and their scores

Groups	Pathological finding	Score
Sham or group (S)	The articular tissue structures including synovium, synovial tissue, adjacent ligaments, tendons and tendinal sheath and subcutaneous tissues and muscles had normal tissue architecture.	0
A day after injected MIA (Inflammation peak day)	Severe acute fibrinopurulent inflammatory reaction. Fibrinopurulent exudates were affected synovial tissues, ligaments, tendons, tendons sheath, muscles and subcutaneous tissues.	4 sharp
Diclofenac gel or group (P)	A very mild edema and presence of a few scattered lymphocytes. Articular tissue structure, including synovium, ligament, tendons, subcutaneous tissue were histologically normal	1
The mixture of extracts or group (T)	As the same as diclofenac.	1
No treatment or group (N)	Chronic inflammatory reactions accompanied by tendinal adhesion were noticed. Formation of granulation tissue with neo vascularization, edema, infiltration of mononuclear leucocytes including lymphocytes and adhesion of tendons to its tendinal sheath were observed.	3

**Fig. 9:**
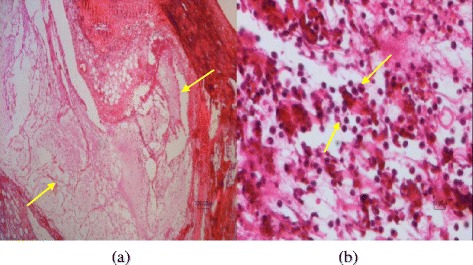
Pathological findings one day after MIA injection. Severe fibrinopurulent exudates (*arrows*) can be seen within articular and adjacent tissues (**a**&**b**). Scale bar =100 μm (**a**), Scale bar =10 μm (**b**)

Rats that received MIA and were euthanized 24 h later showed severe acute fibrinopurulent inflammatory reaction. Fibrinopurulent exudates were affected synovial tissues, ligaments, tendons, tendinal sheath, muscles and subcutaneous tissues (score 4) (Fig. 9).

**Fig. 10:**
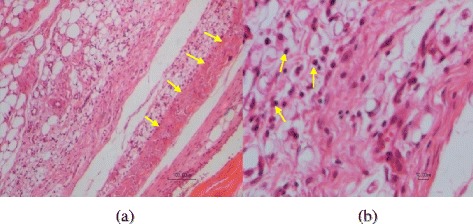
A section of articular tissue from group (P). A very mild reaction including a few scattered lymphoid cells (*arrows*) can be seen. The same tissue reaction was seen in group (T). Scale bar =100 μm (left handed Fig.) and Scale bar =10 μm (right handed Fig.)

In those groups treated by diclofenac gel group (P) or group (T) that received the mixture of the extracts remedy and euthanized 14 days later the same results were obtained. A very mild edema and presence of a few scattered lymphocytes were observed. Articular tissue structure, including synovium, ligament, tendons and subcutaneous tissue were histologically normal (score1) (Fig. 10).

**Fig. 11:**
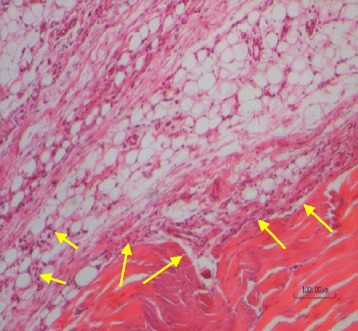
A section of articular tissue from group (N). 14 days after MIA injection, a mixed inflammatory cell infiltration (*small arrows*) and tendinal adhesion to the adjacent tissue (*large arrows*) are evident. Scale bar =100 μm

In the vehicle group that only received MIA and euthanized 14 days later, chronic inflammatory reactions accompanied by tendinal adhesion were noticed. Formation of granulation tissue with neovascularization, edema, infiltration of mononuclear leucocytes including lymphocytes and adhesion of tendons to its tendinal sheath were observed (score 3) (Fig. 11).

According to the obtained data, topical use of the mixture extracts showed anti-inflammatory and analgesic effects on osteoarthritis induced by MIA.

## Discussion

Previous studies have shown that lawsone, isoplumbagin and lawsaritol isolated from *Lawsonia inermis* exhibit anti-inflammatory and analgesic effects in rats [8, 31]. Besides, experiments showed that methanolic extract of *Ricinus communis* leaves, contain flavonoids: rutin, quercetin, epicatechin and polyphenols and gentisic acid have anti-inflammatory activity in rats when administered orally [12] but the topical usage of their combination has not yet been investigated.

Studies showed that intra- articular injection of MIA in rats produces chronic osteoarthritis pain, pharmacological tests were performed in the early (up to 1 week after MIA) versus late (between 2 and 4 weeks after MIA) phase of the rat MIA model [32]. In the present study, six time points 1, 3, 7, 10, 14 and 21 days were taken for determining pain and inflammation to see the effect of the formulation on both acute and chronic phases.

In this study mixture of *Lawsonia inermis* L. and *Ricinus communis* L*.* extracts were used as a topical medication to relief induced joint pain and diclofenac gel which has analgesic and anti-inflammatory effects and has been shown to be effective in the treatment of a variety of acute and chronic pains and inflammatory conditions such as osteoarthritis [33], was used as positive control.

The mixture of extracts showed analgesic effect by reducing mechanical allodynia measured by von Frey filaments increased from a baseline of 0.6 g to 26 g. Comparing the rats of group (N), the significant effect was seen in group (T) after 21 days and in group (P) after 10 days. The responses after day 10, 21 showed that the formulation affected the late inflammatory reactions to painful mechanical stimuli.

We further examined the effects of the mixture using the hot-plate test. The present study has demonstrated the analgesic effect of topical mixture of extracts (0.2 g/0.3 ml) significantly increased the response latency time to heat stimulation after 10 days. This could be the possible explanation for its central analgesic activity observed in hotplate test.

We examined our hypothesis also by using the open field test to exclude false positives in nociceptive tests. The open field test is commonly used for pharmacological selection of drugs that act on the locomotor activities [19]. Rats treated with the mixture of extracts displayed significantly better locomotor recovery at the late stages of the treatment when compared to group (N) after 7 days of MIA injection.

We utilized the differential in weight bearing between the left (osteoarthritic) and right (contralateral control) limbs as an indication of joint discomfort by analysis the rat paw prints. The number and intensity of pixel values of left compared to contralateral paw decreased after 3 days of injection of MIA, same as in group (P).

The acute inflammatory response in the MIA model lasts approximately during the first weak, but afterward inflammation plays a minor role in pain and it is more likely caused by biomechanical forces affecting articular cartilage and subchondral bone [34].

The study showed that topical usage of the mixture of the extracts was useful in the treatment of the inflammation induced by MIA. The effect of reducing inflammation was initiated after 3 days. It showed that the formulation has an effect on acute inflammatory response same as diclofenac gel.

Gross morphological observations and histological evaluation of the knee joints were performed to evaluate the protective effect of the mixture of extracts on cartilage and articular tissue structures. The results of the pathological findings and scoring of the lesions showed that topical usage of the selected formula made significant changes on the knee of the rats histologically. Compared to vehicle group, in which granulation, tissue formation, tendinal adhesion and mixed inflammatory cell infiltration were seen fourteen days after MIA administration, test and diclofenac groups showed only very mild reactions characterized by negligible edema and a few scattered inflammatory lymphoid cells.

According to the pharmacological responses of each herb extract, it could be concluded that the main effect of the formulation related to *L.inermis* extract efficacy.

Although because of the different active compounds in the extracts, the mechanism of action is unknown but the comparable effect on the inhibition of inflammation and pain compared to diclofenac makes the suggestion that they work through the same pathways.

In conclusion, the present study provided clues for further studies on pharmacological methods to analyze the anti-inflammatory and analgesic activities of topical drugs. The results demonstrated that topical preparation of *L.inermis* and *R.communis* was not only be able to mitigate pain and inflammation but also inhibit MIA-induced histological changes on the knee of the rats. Therefore, this formula could be a good candidate for further studies as a new efficient treatment in patients with osteoarthritis.

## Conclusion

This study demonstrated that based on the different anti-inflammatory and analgesic evaluations, the pain and inflammation induced by intra-articular injection of MIA in rats were reduced with topical application of a mixture of *Lawsonia inermis* and *Ricinus communis* extracts. Further clinical studies are required to evaluate the safety and efficacy issues of the extracts mixture.

## Ethic approval

This research was approved by the Research Committee of Shahid Beheshti University of Medical Sciences and institutional ethics committee with approval code SBMU.REC.1392.343.
